# Improvement of the Hydrogen Storage Characteristics of MgH_2_ with Al Nano-Catalyst Produced by the Method of Electric Explosion of Wires

**DOI:** 10.3390/ma17030639

**Published:** 2024-01-28

**Authors:** Viktor N. Kudiiarov, Alan Kenzhiyev, Andrei V. Mostovshchikov

**Affiliations:** Division of Experimental Physics, School of Nuclear Science & Engineering, National Research Tomsk Polytechnic University, Tomsk 634050, Russia; kudiyarov@tpu.ru (V.N.K.); avmost@tpu.ru (A.V.M.)

**Keywords:** magnesium waste, magnesium hydride, aluminum, electric explosion of wires, nanoscale powders, hydrogen, desorption

## Abstract

A new composite with a core–shell structure based on magnesium hydride and finely dispersed aluminum powder with an aluminum oxide shell was mechanically synthesized. We used magnesium chips to produce magnesium hydride and aluminum wire after exploitation to produce nano-sized aluminum powder. The beginning of the hydrogen release from the composite occurred at the temperature of 117 °C. The maximum desorption temperature from the MgH_2_-EEWAl composite (10 wt.%) was 336 °C, compared to pure magnesium hydride—417 °C. The mass content of hydrogen in the composite was 5.5 wt.%. The positive effect of the aluminum powder produced by the electric explosion of wires method on reducing the activation energy of desorption was demonstrated. The composite’s desorption activation energy was found to be 109 ± 1 kJ/mol, while pure magnesium hydride had an activation energy of 161 ± 2 kJ/mol. The results obtained make it possible to expand the possibility of using magnesium and aluminum waste for hydrogen energy.

## 1. Introduction

Magnesium is one of the most common elements on the planet, with a content of about 2% in the earth’s crust. Magnesium is characterized by high specific rigidity and strength, while being light in weight. In this regard, magnesium is used in many industries. The large production and growing consumption of magnesium has led to a greatly increasing volume of magnesium waste, which has extremely negative impacts on the environment. Magnesium waste in the form of chips is regularly generated during the mechanical processing of castings and sheets, while about 30% of magnesium is lost in the form of scrap. In this regard, it is important to resort to magnesium recycling to manage the use of primary magnesium. The hydride dispersion of magnesium waste to produce magnesium hydride is considered as a potential direction for processing magnesium waste. This approach allows for the processing of magnesium waste of any shape and size, without restrictions on quantity, with magnesium hydride as a final product.

Magnesium hydride is one of the most well-known metals, with a hydrogen storage capacity of about 7.6 wt.%. Magnesium proves itself as one of the candidates for its use in hydrogen storage systems. Despite all its advantages, it has a sufficiently high hydrogen yield temperature—about 400 °C due to its high stability, which is reflected in a sufficiently high enthalpy of hydride formation. The microstructure of the material is constantly being improved during high-energy milling [1,2,3], which contributes to obtaining a homogeneous microstructural material [4]. Milling in a planetary ball mill not only improves the microstructure of the material during the milling process, but also destroys the oxide film on the magnesium surface, which allows for accelerating the kinetics of the sorption/desorption reaction.

Further attempts were made to develop suitable catalysts, which led to a rapid absorption and desorption time of hydrogen (2 min at a temperature of 300 °C) for magnesium hydride [5,6,7,8,9,10,11,12,13]. Numerous experiments have been carried out with attempts to change the enthalpy of hydride formation using the chemical composition of the alloy. The most prominent representative is Mg_2_NiH_4_ [14]. In the course of various studies, new methods for the synthesis of magnesium-based hydrides and magnesium hydride with reduced reaction enthalpy and an increased capacity have been developed [15,16,17]. Consequently, many efforts have been made to improve the performance of such hydride-forming metals through a variety of activation techniques and the addition of catalysts.

Several attempts have been made to improve the kinetics of the surface. For this purpose, various additives were used, among which platinum group metals, such as nickel [18,19], hydride-forming Ce, New Brunswick, Ti [20], low-temperature hydrides of the LaNi_5_ type [21,22,23], non-hydride-forming by the type of iron, cobalt, and chromium, positively affecting the kinetics of the sorption of magnesium-based hydrides [24,25], as well as metal oxides [8]. Three-dimensional transition metalloids and oxides could change and significantly improve the sorption kinetics [9,10,13,26,27,28,29,30,31].

The most promising of the known metals, mentioned before, for hydrogen storage in the bound state is magnesium [32,33,34,35,36]. It is most commonly used in various studies, observing its interaction with hydrogen and improving its sorption and desorption characteristics with various catalysts. Many studies have considered and investigated various metals as additives such as nickel [37,38], cobalt [39], iron [40], titanium [41], vanadium [42], copper [43], palladium [44], fluorides, and so on [45,46,47,48,49,50,51].

All the studies were aimed at establishing the nature of the interaction of magnesium with hydrogen in the presence of the above catalysts. Despite this variety of studies, the problem of the high stability of the metal–hydride bond and, consequently, the high temperature of hydrogen desorption from the material remains relevant to this day. At the same time, in similar studies [34,38,42,52,53,54], the question of finding the optimal ratio between the acceptable hydrogen yield temperature and the maximum hydrogen content by mass often arises. Also, the size of the added particles plays an important role, and could lead to achieving the desired result. The particle size factor of added catalyst powders plays a key role in the process of hydrogen desorption from magnesium hydride and affects the process temperature. The choice of the optimal catalyst particle size is based on the required desorption kinetics and the uniformity of catalyst distribution over the material volume. To improve the efficiency of the process of hydrogen desorption from magnesium hydride, researchers are studying various ways to modify this material. One possible solution is the addition of aluminum. Aluminum has a number of properties that make it attractive for use as an additive to magnesium hydride. First, it is highly active in reactions with hydrogen, which can accelerate the process of hydrogen release from MgH_2_. Secondly, aluminum has a low cost and availability, making it a cost-effective option for industrial-scale use. One of the main issues associated with the addition of aluminum to magnesium hydride is the effect of this process on the hydrogen desorption temperature. Since magnesium hydride has a high stability of hydrogen bonds, i.e., a high desorption temperature, there are some solutions to reduce it and, consequently, to reduce the activation energy of desorption: additional grinding in a planetary ball mill, the addition of catalysts, varying the synthesis parameters, etc.

In the study of Varin R. et al. [54], nanocrystalline magnesium–nickel hydride MgH_2_, the grains (crystallites) of which have a size of about 11–12 nm, was used. It was synthesized in a magnetic mill by the controlled mechanical milling of magnesium powder with the addition of various forms of nickel–lead powder obtained through the decomposition of nickel carbonyl from 0.5 to 2 wt%. The powder catalysts used were designated as micro-Ni, submicro-Ni, and nano-Ni, respectively. Based on the data obtained, nano-Ni was found to be the most effective catalyst with the property of significantly increasing the rate of hydrogen uptake by magnesium to form hydride. At an addition of 0.5 wt% of nano-nickel catalyst powder, the rate of hydrogen absorption increased approximately twice in comparison with pure magnesium.

The LiMg(AlH_4_)_3_ hydride used in the study [55] is able to release about 7.2 wt.% of the total amount of hydrogen during the two-step thermal decomposition reaction. The authors indicate that the first temperature peak was observed at 120 degrees Celsius and the second at 160 degrees Celsius. The decomposition process of MgD2 is explained by thermodynamic destabilization due to the formation of Al_x_Mg_y_ phases. The experimentally detected decomposition modes of Li-Mg alanate are presumably related to the enthalpy of decomposition, since the contaminated or accelerated LiMgAlH_6_ intermediate may participate more actively in the thermal decomposition reaction at lower temperatures, whereas pure LiMgAlH_6_, which has kinetic barriers, requires higher temperatures to initiate the two-step decay during the exothermic reaction.

Lyu et al. [56] evaluated the hydrogen accumulation properties of Mg and Mg-Al hydrides. The authors found several key points in their study: a 10 at.% aluminum coating accelerates hydrogen desorption by a factor of 10 and has a higher hydrogen content compared to Mg coating, and Mg-10 at.% Al exhibits a temperature decrease of 17 degrees Celsius. The temperature decrease is due to the weakening of the Mg-H bond. In the study, the authors also suggested that this behavior of the aluminum substrate is due to the discontinuous growth on the polyimide base. Aluminum forms island-like islands that do not form an alloy with magnesium and serve as heterogeneous nucleation centers to trap hydrogen atoms.

Shang et al. [57] demonstrated, in their study, powder mixtures based on magnesium hydride with doping of 8 mol% aluminum, nickel, iron, titanium, copper, and niobium. According to the results, the heat of the formation obtained during thermally stimulated desorption was at 300 °C, and the MgH_2_-8 mol% Al system had a heat of formation −28.36 kJ/mol, compared to magnesium hydride −75.99 kJ/mol. The extent of the effect of increasing the desorption rate and interaction with hydrogen decreases from Ni, Al, Fe, Nb, and Ti to Cu.

Despite the large number of studies in the field of hydrogen energy, theoretical calculations, such as density functional theory or computer modeling, remain an important aspect. Relatively few such works have been carried out. Due to the complexity of such processes, the authors Gu et al. carried out calculations using path integral formalism and the polymer ring model in the density functional theory. The results of their calculations using the density functional theory in combination with the theory of Rosenfeld measures allowed them to describe the nature of adsorption and provided an opportunity for further ground for research in this area [58].

In [59], the authors Vostrikov et al. [59] demonstrated the efficiency of liquid organic hydrogen carriers. The authors suggest that this method of hydrogen storage is safe and has a denser form due to the peculiarities of covalent bonds. Sorption and desorption in such material was carried out by catalytic process. One of the most important, according to the authors, in this work was the enthalpy of the reaction, which determines the amount of heat in the desorption of hydrogen, as well as the equilibrium of the reaction. The work combines experimental, quantum, and additive approaches, which allowed a detailed study of reaction enthalpies. The main aspect of the work was the demonstration of the positive influence of the number and position of methyl groups on the enthalpies of reactions. Thus, in this work, the lowest enthalpy value in the dehydrogenation reaction was observed for the five-membered ring with the addition of a nitrogen heteroatom.

The authors Alshahrie et al., [60] as well as others, were puzzled by the question of finding an effective material for hydrogen storage. In this connection, they considered a pseudocapsule material based on metallic glass for the electrochemical storage of hydrogen. The material-storage in this work has a certain prospect of use due to its calculated capacity, according to the authors of the work—950 mAh/g. Also, about 40 cycles were carried out with the calculation of capacity preservation, which was 97%. The high-speed discharge and capacity retention allow us to speak about the high stability of the Ni_60_Pd_20_P_16_B_4_ compound. 

On this basis, a number of studies have been carried out to determine the effect of the size of the added particles on the kinetics of sorption and desorption reactions. The same was carried out in this study—nano-sized aluminum powder produced by the method of the electric explosion of wires (EEW) was used and its effect on the hydrogen yield temperature from the composite was determined. In this work, EEW-aluminum powder obtained on the basis of the Tomsk Polytechnic University acts as such an additive [56,57]. The EEW method for producing nanoscale powders, in fact, combines the characteristics of dispersing methods—the destruction of the conductor under the action of electricity and methods of vaporization and condensation— and most of the material of the conductor during the electric pulse explosion passes into the gas phase. And in this case, the amount of metal which is turned into vapor is determined by the amount of energy injected into the conductor. One of the advantages of electric explosive technology is its flexibility—it allows one to produce nanoscale powders of metals, alloys, intermetallides, and chemical compounds with nonmetals using the same equipment. In the production of nanoscale powders of metals, alloys, and intermetallic substances, inert gases, especially argon, are usually used as a gas medium. Nanoscale powders produced in such an environment are pyrophoric, i.e., they easily burn in contact with oxygen. To prevent this effect, they are passivated by slow oxidation with air components or by applying a special coating to their surface. It is important to note that the properties of nanoscale powders produced by the electrical explosion of wires (EEW) method strongly depend not only on the electrical parameters of their production, but also on the conditions of passivation.

The method of EEW is widely used to obtain nano-sized metal powders, including from wires after their operation. This powder has a core–metal structure and the shell is an oxide film, which allows it to be used to improve the properties of a composite based on magnesium hydride. Thus, the aim of this research is to study the microstructure of a magnesium hydride-based composite with an aluminum powder addition produced by an electric wire explosion and its characteristics of interaction with hydrogen for further use in the field of hydrogen energy.

## 2. Materials and Methods

In this work, magnesium hydride powder was obtained by the method of hydride dispersion of pure (99%) magnesium chips. Particle size of obtained powder is up to 300 microns. Fine aluminum powder was produced from aluminum wire after operation by the method of electric explosion of wires. The Division of Experimental Physics at Tomsk Polytechnic University developed a Gas Reaction Automated Machine (GRAM) to prepare magnesium hydride and study the accumulation of hydrogen in it.

An initial activation of magnesium was carried out using a planetary ball mill to increase its surface area and improve its interaction with hydrogen in the GRAM complex. The ratio of the mass of balls to the mass of magnesium powder in milling jars was 10:1. Synthesis of the samples was also carried out using this equipment for 2 h in argon atmosphere with a ball-to-powder mass ratio of 20:1. All parameters were selected on the basis of previous studies, including those carried out at the Division of Experimental Physics by our research group.

Preparation of samples and their loading into milling jars was carried out in argon atmosphere (99.9% purity) using a leak-proof glove box SPEKS GB 02M (“Spectroscopic Systems”, Moscow, Russia). The mass content of hydrogen was analyzed using RHEN602 instrument (LECO Corporation, St. Joseph, MI, USA). The microstructure of the obtained materials was investigated using a TESCAN VEGA 3 SBU microscope (Tescan Orsay Holding a.s., Brno, Czech Republic), and the elemental composition was analyzed by energy dispersive spectroscopy (X-Max 50, Oxford Instruments PLC, Abingdon, UK). The phase composition of the materials was studied by X-ray diffraction in the angle range of 5–90° (XRD-7000S, Shimadzu, Tokyo, Japan). High-resolution transmission electron microscopy (HRTEM) images were obtained using JEM-2100F (JEOL, Tokyo, Japan).

Thermal Stimulated Desorption (TSD) is an experimental technique used to study the properties of materials, particularly composites. In the process of thermally stimulated desorption, the sample is heated, which promotes the desorption (release) of gases that have been absorbed by the material while it has been exposed to a specific environment. The basic principle of the method is that the gases absorbed by the material are released from heating temperatures, allowing their characteristics to be studied and their concentration quantified. This provides information on the absorption of gases, the structure of the material, its chemical stability, and the effect of various influences on its properties.

## 3. Results and Discussions

**Figure 1:**
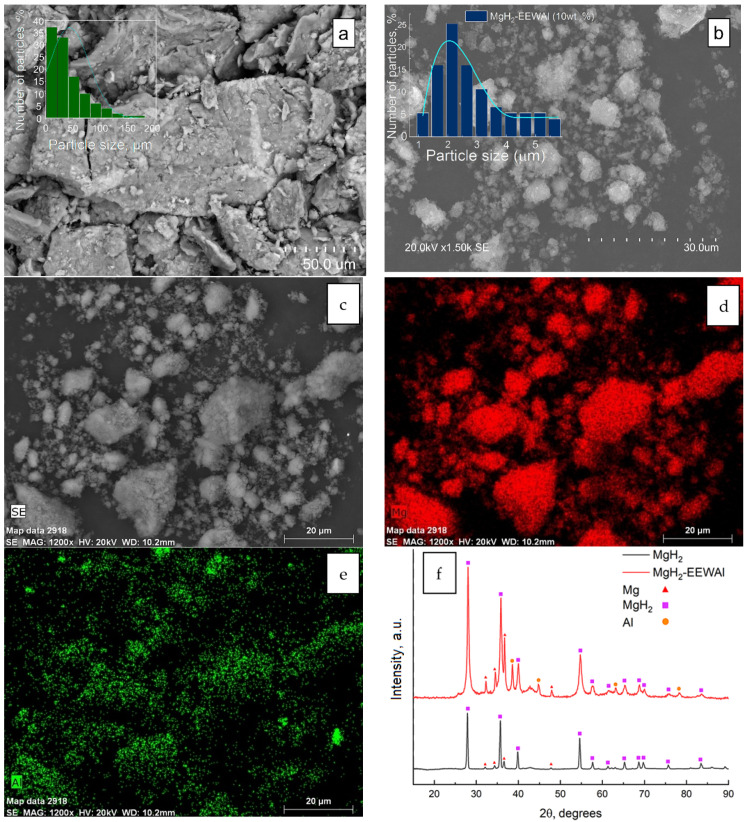
SEM micrographs of MgH_2_ (**a**), composite MgH_2_-10 wt.% EEWAl (**b**), and particle size distribution of composite. EDX distribution maps of Mg and Al (**c**–**e**) and XRD patterns of magnesium hydride and composite MgH_2_-EEWAl (10 wt.%) (**f**).

The microstructure and X-ray diffraction patterns of magnesium hydride and its composite are shown in Figure 1. The obtained experimental data indicate a positive effect in the reduction of the particle size in the synthesis process in a planetary ball mill. A change in the microstructure is demonstrated, which in turn, based on previous studies, also had a positive effect on the desorption characteristics of the material. The energy dispersive analysis shown in Figure 1c–e allowed us to create distribution maps of elements on the surface of the sample. During this analysis, the scattered X-rays recorded when the sample was exposed to the electron beam allowed the identification of the elements present in the material. Elemental mapping during energy dispersive analysis provided information on the micro- and nanostructure of the material, its chemical composition, and the spatial distribution of elements on the sample surface.

X-ray diffraction analysis on the sample surface allowed the identification of the phases present in the materials. Diffraction patterns of the materials are typical for the obtained samples. The observed phases are mainly magnesium hydride, magnesium, and aluminum phases. The synthesis of the materials resulted in an increase in the micro strains of the material, which is a consequence of the method used to produce the composite.

After obtaining samples by scanning electron microscopy, the size of the magnesium hydride particles was found to be about 150 μm with agglomerates reaching about 200 μm in size (Figure 1a). The selected rotational speed of the grinding bowls allowed the obtainment of magnesium hydride particles of a smaller size. Figure 1f clearly shows that most of the phase composition is magnesium hydride with a small amount of magnesium phase. Particle size reduction using a planetary ball mill is an important process in the field of materials science. The benefits of particle size reduction using a planetary ball mill include the ability to produce materials with improved physical, chemical, and mechanical properties by increasing the specific surface area of the particles, as well as creating nanostructured materials with unique properties.

A scanning electron microscopy study showed a significant decrease in the size of the composite particles up to 11 µm (Figure 1b), which indicates the optimally selected parameters of the synthesis of the materials—900 rpm for 2 h. The elemental composition of the composite is mainly magnesium hydride with uniformly distributed EEW aluminum powder particles on magnesium (Figure 1c–e). At the same time, an analysis of the phase composition of the materials did not reveal the presence of extraneous elements. The diffraction patterns show characteristic peaks observed at appropriate diffraction angles in the range of angles of 5–90°. 

**Figure 2:**
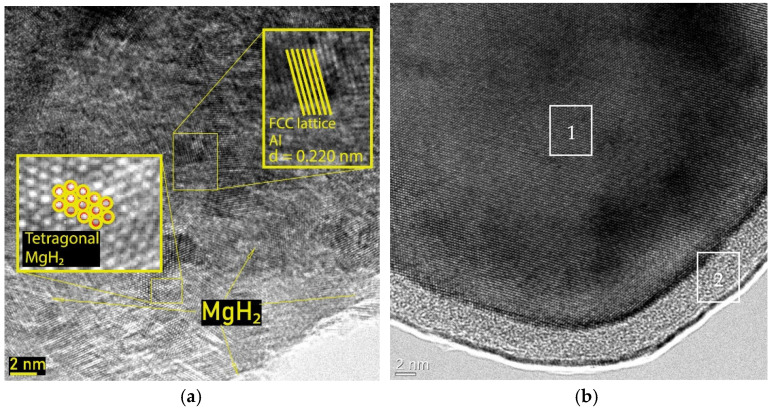
HRTEM micrographs (**a**) of the MgH_2_-EEWAl sample (10 wt.%); EEWAl (**b**) TEM micrography (1—Al core, 2—oxide shell).

HRTEM micrographs of the MgH_2_-EEWAl sample (10 wt.%) and separate particles of Al are shown in the Figure 2. The method of transmission electron microscopy allowed us to specify the crystal structures of the synthesized materials.

The structure of the composite obtained through synthesis in a planetary ball mill is shown in Figure 2a and it can be observed how the aluminum and magnesium hydride atoms are distributed in the volume. Aluminum has an FCC lattice while magnesium hydride has a tetragonal rutile-type lattice. According to microscopy data, the aluminum nanoparticle (Figure 2b) is a homogeneous metal core 1, covered with a homogeneous protective passivating oxide–hydroxide shell 2. The figure clearly shows the structures of the components of this composite. The interplane distance for aluminum was experimentally obtained equal to 0.220 nm (theoretical 0.202 nm). Presumably, this structure of magnesium hydride and aluminum hydride allows for weaker Mg-H bonds and promotes the faster decomposition of magnesium hydride. 

Further, the volumetric and structural characteristics of the material were evaluated using BET surface analysis. The method proposed by Brunauer, Emmett, and Teller (BET) is a widely used method for determining the specific surface area of materials. This method is based on measuring the volume of gas absorbed by a material at different relative pressures and then analyzing this data using the Brunauer–Emmett–Teller equation. The BET equation describes the relationship between the volume of gas absorbed and the relative pressure in the pores of the material. It takes into account that gas molecules will preferentially adsorb on the pore surface of the material, resulting in an increase in the volume of absorbed gas as the relative pressure increases. Using the BET equation, the specific surface area of the material can be calculated based on measured data on the volume of absorbed gas at different relative pressures. This parameter is an important parameter because the specific surface area of a material affects its chemical, physical, and technological properties. The BET method provides information on the structure of porous materials, their surface area, and properties, which is important for the development of new materials and the optimization of production processes.

**Figure 3:**
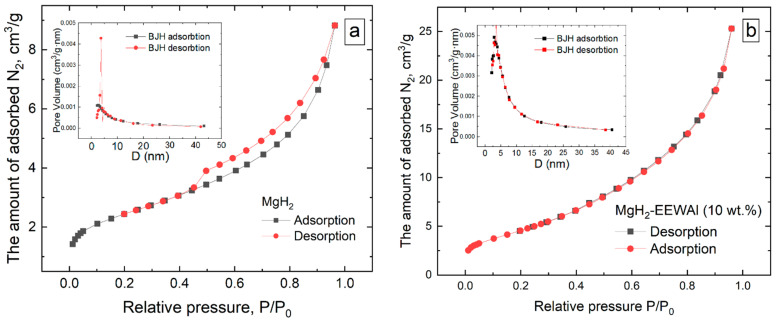
BET absorption and desorption curves at 77 K with pore size distribution (PSD) for MgH_2_ (**a**) and MgH_2_-EEWAl (**b**) composite.

As mentioned earlier in the text, the additional milling process in the planetary ball mill helped to significantly increase the surface area of the materials in order to improve their interaction with hydrogen, for which a corresponding BET analysis was performed to confirm this statement (Figure 3).

During the BET analysis, it was found that the surface area was increased from 8.5 to 17.5 m^2^/g. The co-milling of magnesium and aluminum hydride particles in a planetary ball mill is a process of the mechanical milling of materials under the action of friction and impact forces. This process results in a reduction in the particle size and an increase in the particle surface area. Magnesium hydride and aluminum are highly reactive, and grinding these materials together in the mill results in an increase in the surface area of each particle. This in turn increases the availability of active sites for chemical reactions such as hydrogenation or oxidation. Increasing the surface area of magnesium and aluminum hydride particles can be useful in various industrial processes such as the production of composite materials, catalysts, or energy-intensive systems. 

**Figure 4:**
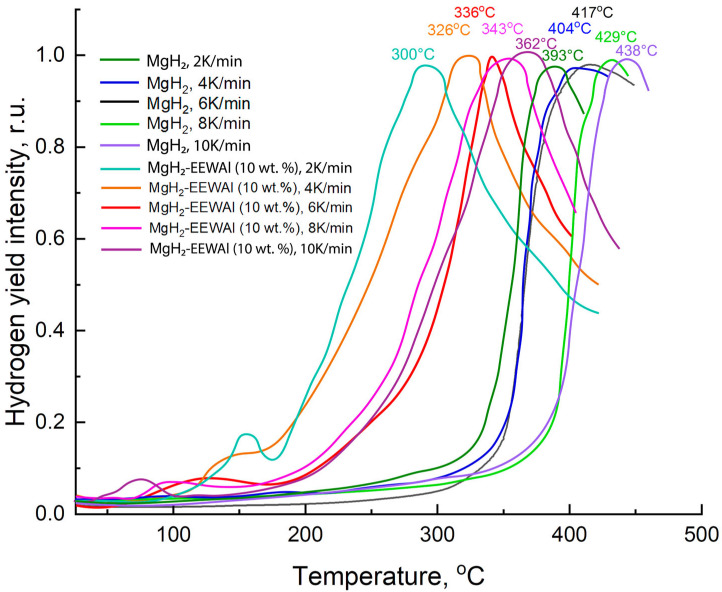
Comparison of TSD curves for MgH_2_ and composite MgH_2_-EEWAl (10 wt.%).

According to the results of the thermally stimulated desorption (TSD) (Figure 4), it was demonstrated that the largest amount of hydrogen released was at a temperature of about 336 °C at a heating rate of 6 K/min. In comparison with pure magnesium hydride, the difference in the desorption temperatures is 81 degrees. This decrease in temperature is presumably due to its structural and phase changes during the co-milling process in a planetary ball mill, as a consequence of the catalytic effect of aluminum and its ability to destabilize Mg-H bonds.

Presumably, the decrease in the desorption temperature was influenced by the catalytic effect of the addition of aluminum powder produced by the electric explosion of wires method. At the same time, the temperature at the beginning of the hydrogen release turned out to be about 117 °C, and the maximum was 336 °C. There was also an effect from the joint milling of the synthesized materials, which made it possible to reduce the particle size and thereby reduce the activation energy of desorption. Based on the results of thermally stimulated desorption, the desorption activation energy was calculated, presented below. The Kissinger method is an overwhelmingly popular way of estimating the activation energy of thermally stimulated processes. The essence of the method is to remove these desorption curves at different heating rates (in this case, 2, 4, 6, 8, and 10 K/min), resulting in peak shifts. The temperature values at peaks at different heating rates are taken, a calculation is performed to construct in Arrhenius coordinates (1000/*T_p_* from *ln(β/T*^2^*_p_*)), and the angular coefficient (*A*) is determined, which gives the activation energy (*E_d_*). 

**Figure 5:**
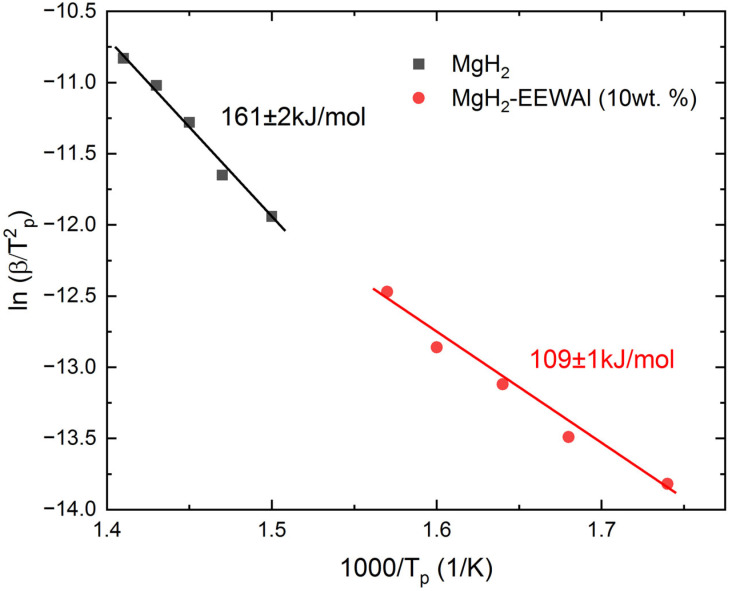
$\frac{1000}{T_{p}}$ from $ln\frac{\beta }{T_{P}^{2}}$ dependency graphs of pure MgH_2_ and composite MgH_2_-EEWAl (10 wt.%).

Based on the obtained values of the temperature maxima at different heating rates, a recalculation was performed for the construction in Arrhenius coordinates (Figure 5) and the angular coefficient *A* was found, which could then be applied in calculating the activation energy.

(1) $$ln\frac{\beta }{T_{P}^{2}}=A-\frac{E_{d}}{RT_{p}},$$

where *A* is the angular coefficient, *R* is the universal gas constant, *β* is the heating rate, and *T_p_* is the temperature of the peak hydrogen yield.

Based on the above equation, it becomes possible to determine the activation energy of desorption. Further, Arrhenius curves were constructed (Figure 5), which also allowed us to estimate this energy.

The data obtained show a significant decrease in the activation energy of desorption, which is due to the catalytic effect of the addition of aluminum obtained by the electric wire explosion method.

According to the calculation results, it is clearly showed that there is a decrease in the energy. The difference between the initial magnesium hydride and the composite is 52 kJ/mol. 

In similar works [61,62], the authors demonstrated a positive effect in reducing the desorption activation energy by adding MIL-101 (Cr). The desorption activation energy for the MgH_2_-EEWAl (10 wt.%) composite was 109 kJ/mol, while in [61,62], it was 120 kJ/mol.

In a similar study by the authors Huaigang et al. [63], the activation energy for HZSM-5 zeolite was determined to be of the order of 54 kJ/mol. The authors attribute this decrease to the mechanism of HSZM-5 zwitterion, which acted as an amine adsorbent and catalyst.

Fedorov et al., in [64], demonstrated that the data obtained by the Kissenger method for the reduction of copper oxide in CuO-NiO, compared to pure copper oxide, showed the activation energies increase from 38 to 54 kJ/mol, respectively. According to the authors, this is due to the formation of Ni solid solution in CuO, which in turn leads to the nucleation of nucleation sites.

Presumably, nanoscale particles of aluminum produced by the electric explosion of wires method can reduce the activation energy due to joint milling in a planetary ball mill and changes in the structure of magnesium hydride itself. This achieves a catalytic and synergistic effect in reducing the desorption temperature by 100 degrees. The shift of the temperature peak of hydrogen desorption from magnesium hydride is presumably related to structural and phase changes in the obtained composite during co-milling. The catalytic effect that is shown in this work is a base for further studies related to this type of additive. In the future, it is planned to vary the fraction of grinding balls, synthesis parameters, and the amount of added material to establish the regularities of composite behavior. 

## 4. Conclusions

In this article, we used magnesium chips to produce magnesium hydride and aluminum wire after use to produce nano-sized aluminum powder. The composite MgH_2_-10 wt.%EEWAl was synthesized by ball milling using obtained magnesium hydride and aluminum nano-sized powder. The microstructure and hydrogenation/dehydrogenation properties of the composite MgH_2_-10 wt.%EEWAl and the effect of EEWAl on the hydrogen properties of Mg/MgH_2_ were determined. Using scanning electron microscopy, it was shown that the composite consists of MgH_2_ particles with a uniform distribution of aluminum nanoparticles on their surface. The average particle size of the composite is about 1.5 microns. Based on the experimental results, the addition of nanoscale aluminum powder improved the desorption characteristics of MgH_2_. The addition of aluminum powder produced by the electric explosion of wires method made it possible to reduce the desorption temperature to 336 °C at a heating rate of 6 K/min in comparison with conventional magnesium hydride, the hydrogen yield temperature of which was 417 °C. This is presumably due to the catalytic effect in reducing the activation energy (ΔE_d_ = 52 kJ/mol) of desorption, which in turn led to a significant reduction in the temperature. The beginning of the hydrogen release occurred at a temperature of about 117 °C. The mass content of hydrogen in the composite was 5.5 wt.%. The results obtained make it possible to expand the possibility of using magnesium and aluminum waste for hydrogen energy.

## Data Availability

The raw/processed data required to reproduce these findings cannot be shared at this time as the data also forms part of an ongoing study.
